# Supplementary material for: Hot spot prediction in protein-protein interactions by an ensemble system
Source: BMC Syst Biol. 2018 Dec 31;12(Suppl 9):132. doi: 10.1186/s12918-018-0665-8 (PMC6311905; doi:10.1186/s12918-018-0665-8)
Supplement: Supplementary file 2 — All relevant MATLAB code. A simple Matlab implement of our predictor. (ZIP 19,742 kb) [file 12918_2018_665_MOESM2_ESM.zip › Additional file 2/Read ASA/ASA website/SKEMPI/1MAHA.htm]

 NetSurfP - Protein Surface Accessibility and Secondary Structure Predictions

  

|  |  |  |
| --- | --- | --- |
|  |  | NetSurfP - Protein Surface Accessibility and Secondary Structure Predictions  Technical University of Denmark |

  


---

```
# For publication of results, please cite:
# A generic method for assignment of reliability scores applied to solvent accessibility predictions.
# Bent Petersen, Thomas Nordahl Petersen, Pernille Andersen, Morten Nielsen and Claus Lundegaard
# BMC Structural Biology 2009, 9:51 doi:10.1186/1472-6807-9-51
#
# Column 1: Class assignment - B for buried or E for Exposed - Threshold: 25% exposure, but not based on RSA
# Column 2: Amino acid
# Column 3: Sequence name
# Column 4: Amino acid number
# Column 5: Relative Surface Accessibility - RSA
# Column 6: Absolute Surface Accessibility
# Column 7: Z-fit score for RSA prediction
# Column 8: Probability for Alpha-Helix
# Column 9: Probability for Beta-strand
# Column 10: Probability for Coil
E E  1MAHA                  1    0.931 162.698   0.283   0.003   0.003   0.994
E D  1MAHA                  2    0.741 106.793  -0.551   0.005   0.015   0.979
E P  1MAHA                  3    0.617  87.496  -1.636   0.005   0.045   0.951
E Q  1MAHA                  4    0.557  99.480   1.028   0.019   0.141   0.840
B L  1MAHA                  5    0.098  17.980  -0.159   0.004   0.616   0.381
E L  1MAHA                  6    0.304  55.699   1.497   0.001   0.959   0.040
B V  1MAHA                  7    0.056   8.653   0.685   0.000   0.983   0.017
E R  1MAHA                  8    0.394  90.203   1.349   0.001   0.959   0.040
B V  1MAHA                  9    0.038   5.794   0.073   0.002   0.816   0.182
E R  1MAHA                 10    0.657 150.384  -0.039   0.004   0.197   0.799
E G  1MAHA                 11    0.575  45.237  -0.523   0.005   0.015   0.979
B G  1MAHA                 12    0.082   6.469  -1.198   0.004   0.514   0.481
E Q  1MAHA                 13    0.315  56.313   0.993   0.001   0.900   0.099
B L  1MAHA                 14    0.032   5.951  -0.562   0.001   0.959   0.040
E R  1MAHA                 15    0.238  54.502   1.686   0.001   0.959   0.040
B G  1MAHA                 16    0.036   2.865  -2.515   0.001   0.959   0.040
E I  1MAHA                 17    0.231  42.735   0.791   0.001   0.959   0.040
B R  1MAHA                 18    0.241  55.097   0.616   0.001   0.959   0.040
E L  1MAHA                 19    0.259  47.405   0.710   0.001   0.900   0.099
E K  1MAHA                 20    0.481  98.983  -0.690   0.003   0.718   0.279
B A  1MAHA                 21    0.268  29.512  -1.118   0.005   0.336   0.660
E P  1MAHA                 22    0.627  88.943  -1.368   0.004   0.085   0.910
E G  1MAHA                 23    0.729  57.341  -1.611   0.005   0.015   0.979
E G  1MAHA                 24    0.516  40.633  -0.265   0.005   0.015   0.979
E P  1MAHA                 25    0.382  54.177   0.756   0.005   0.336   0.660
B V  1MAHA                 26    0.076  11.697   0.655   0.002   0.816   0.182
B S  1MAHA                 27    0.191  22.373   0.745   0.011   0.918   0.071
B A  1MAHA                 28    0.214  23.561   1.057   0.011   0.918   0.071
B F  1MAHA                 29    0.066  13.206  -0.660   0.001   0.900   0.099
B L  1MAHA                 30    0.248  45.482   0.569   0.004   0.514   0.481
B G  1MAHA                 31    0.305  23.988  -1.133   0.004   0.197   0.799
B I  1MAHA                 32    0.065  11.951   1.210   0.019   0.141   0.840
E P  1MAHA                 33    0.287  40.697   0.025   0.020   0.205   0.775
B F  1MAHA                 34    0.163  32.734  -0.678   0.019   0.141   0.840
B A  1MAHA                 35    0.055   6.050  -0.575   0.056   0.142   0.802
E E  1MAHA                 36    0.527  91.980   1.002   0.018   0.088   0.893
B P  1MAHA                 37    0.222  31.473   0.396   0.018   0.047   0.935
B P  1MAHA                 38    0.302  42.854  -0.657   0.018   0.047   0.935
E V  1MAHA                 39    0.391  60.112  -0.733   0.058   0.017   0.925
E G  1MAHA                 40    0.426  33.526  -0.584   0.058   0.017   0.925
E S  1MAHA                 41    0.751  88.005   0.877   0.115   0.016   0.868
B R  1MAHA                 42    0.267  61.235   0.249   0.113   0.043   0.844
B R  1MAHA                 43    0.207  47.517   0.429   0.053   0.043   0.903
B F  1MAHA                 44    0.098  19.689  -0.397   0.052   0.084   0.864
E M  1MAHA                 45    0.453  90.665   1.079   0.052   0.084   0.864
E P  1MAHA                 46    0.461  65.458   0.630   0.018   0.047   0.935
B P  1MAHA                 47    0.112  15.907  -0.954   0.018   0.047   0.935
E E  1MAHA                 48    0.445  77.654   0.173   0.018   0.088   0.893
E P  1MAHA                 49    0.498  70.723   0.328   0.018   0.047   0.935
E K  1MAHA                 50    0.272  55.992   0.220   0.018   0.047   0.935
E R  1MAHA                 51    0.662 151.621  -0.379   0.018   0.019   0.964
E P  1MAHA                 52    0.513  72.795  -0.328   0.018   0.019   0.964
B W  1MAHA                 53    0.170  40.837  -1.546   0.018   0.019   0.964
E S  1MAHA                 54    0.680  79.719  -0.142   0.058   0.017   0.925
E G  1MAHA                 55    0.409  32.157  -0.346   0.052   0.084   0.864
B V  1MAHA                 56    0.272  41.745  -0.359   0.021   0.451   0.528
B L  1MAHA                 57    0.227  41.582   0.745   0.023   0.655   0.322
E D  1MAHA                 58    0.322  46.386   0.111   0.022   0.552   0.426
B A  1MAHA                 59    0.025   2.722  -0.620   0.066   0.296   0.638
E T  1MAHA                 60    0.388  53.774  -0.254   0.056   0.142   0.802
E T  1MAHA                 61    0.544  75.508  -0.519   0.056   0.142   0.802
E F  1MAHA                 62    0.342  68.700  -0.773   0.018   0.047   0.935
E Q  1MAHA                 63    0.403  71.994  -1.524   0.005   0.015   0.979
E N  1MAHA                 64    0.410  60.009  -1.462   0.005   0.015   0.979
B V  1MAHA                 65    0.274  42.083  -0.725   0.018   0.047   0.935
B C  1MAHA                 66    0.023   3.299  -0.435   0.018   0.047   0.935
B Y  1MAHA                 67    0.101  21.605  -0.816   0.018   0.088   0.893
B Q  1MAHA                 68    0.057  10.109  -1.249   0.018   0.088   0.893
E Y  1MAHA                 69    0.344  73.491  -0.515   0.018   0.047   0.935
E V  1MAHA                 70    0.373  57.392  -1.521   0.058   0.017   0.925
E D  1MAHA                 71    0.556  80.192  -2.117   0.058   0.017   0.925
E T  1MAHA                 72    0.414  57.477  -2.417   0.115   0.016   0.868
E L  1MAHA                 73    0.382  70.036  -1.831   0.113   0.043   0.844
E Y  1MAHA                 74    0.335  71.590  -1.833   0.184   0.043   0.773
E P  1MAHA                 75    0.404  57.328  -2.263   0.181   0.016   0.803
E G  1MAHA                 76    0.345  27.128  -2.064   0.184   0.043   0.773
B F  1MAHA                 77    0.250  50.155  -2.022   0.268   0.043   0.689
E E  1MAHA                 78    0.356  62.123  -1.922   0.268   0.043   0.689
E G  1MAHA                 79    0.304  23.941  -2.537   0.268   0.043   0.689
B T  1MAHA                 80    0.178  24.730  -2.028   0.268   0.043   0.689
E E  1MAHA                 81    0.355  61.931  -1.385   0.191   0.086   0.723
B M  1MAHA                 82    0.286  57.289  -2.035   0.191   0.086   0.723
E W  1MAHA                 83    0.374  89.923  -1.767   0.191   0.086   0.723
E N  1MAHA                 84    0.413  60.449  -1.751   0.113   0.087   0.800
E P  1MAHA                 85    0.553  78.457  -1.831   0.053   0.043   0.903
E N  1MAHA                 86    0.546  79.890  -1.852   0.052   0.084   0.864
E R  1MAHA                 87    0.335  76.830  -0.820   0.019   0.141   0.840
E E  1MAHA                 88    0.479  83.751  -0.856   0.020   0.205   0.775
E L  1MAHA                 89    0.298  54.637  -1.428   0.020   0.205   0.775
B S  1MAHA                 90    0.194  22.737  -1.616   0.019   0.141   0.840
B E  1MAHA                 91    0.305  53.301  -0.570   0.018   0.088   0.893
E D  1MAHA                 92    0.293  42.236   0.152   0.004   0.138   0.858
B C  1MAHA                 93    0.031   4.310   0.487   0.004   0.514   0.481
B L  1MAHA                 94    0.027   4.944   0.327   0.002   0.816   0.182
B Y  1MAHA                 95    0.086  18.442   0.117   0.001   0.959   0.040
B L  1MAHA                 96    0.019   3.387   1.303   0.001   0.959   0.040
B N  1MAHA                 97    0.035   5.065  -0.117   0.001   0.900   0.099
B V  1MAHA                 98    0.020   3.074   0.769   0.001   0.900   0.099
B W  1MAHA                 99    0.074  17.725  -0.832   0.002   0.816   0.182
B T  1MAHA                100    0.090  12.483   0.751   0.004   0.514   0.481
B P  1MAHA                101    0.064   9.067  -0.251   0.005   0.262   0.733
E Y  1MAHA                102    0.522 111.637   0.208   0.005   0.045   0.951
E P  1MAHA                103    0.488  69.205  -0.522   0.018   0.019   0.964
E R  1MAHA                104    0.522 119.515   0.071   0.005   0.015   0.979
E P  1MAHA                105    0.745 105.659  -1.410   0.003   0.003   0.994
E A  1MAHA                106    0.796  87.686  -0.996   0.003   0.003   0.994
E S  1MAHA                107    0.536  62.796   0.584   0.003   0.003   0.994
E P  1MAHA                108    0.432  61.287   0.967   0.005   0.015   0.979
B T  1MAHA                109    0.089  12.400   1.322   0.004   0.138   0.858
B P  1MAHA                110    0.034   4.839   0.237   0.004   0.514   0.481
B V  1MAHA                111    0.019   2.951   1.031   0.001   0.900   0.099
B L  1MAHA                112    0.019   3.406   1.544   0.001   0.959   0.040
B I  1MAHA                113    0.014   2.535   1.533   0.001   0.959   0.040
B W  1MAHA                114    0.023   5.652   1.163   0.001   0.959   0.040
B I  1MAHA                115    0.018   3.404   1.175   0.001   0.900   0.099
B Y  1MAHA                116    0.056  11.903   0.824   0.004   0.420   0.576
B G  1MAHA                117    0.049   3.864  -1.371   0.018   0.047   0.935
B G  1MAHA                118    0.081   6.375  -1.616   0.058   0.017   0.925
B G  1MAHA                119    0.081   6.343  -2.064   0.052   0.084   0.864
B F  1MAHA                120    0.075  15.093  -0.020   0.064   0.216   0.721
B Y  1MAHA                121    0.136  29.106  -0.765   0.066   0.296   0.638
B S  1MAHA                122    0.223  26.112  -0.833   0.021   0.279   0.699
B G  1MAHA                123    0.079   6.241  -3.803   0.020   0.205   0.775
B A  1MAHA                124    0.132  14.601  -2.053   0.019   0.141   0.840
B A  1MAHA                125    0.166  18.249  -3.020   0.018   0.047   0.935
E S  1MAHA                126    0.344  40.305  -1.709   0.018   0.047   0.935
B L  1MAHA                127    0.270  49.419  -1.234   0.018   0.019   0.964
E D  1MAHA                128    0.382  55.032  -1.578   0.018   0.047   0.935
B V  1MAHA                129    0.287  44.158  -2.241   0.018   0.088   0.893
B Y  1MAHA                130    0.104  22.246  -1.247   0.018   0.088   0.893
E D  1MAHA                131    0.303  43.720  -0.569   0.018   0.047   0.935
B G  1MAHA                132    0.105   8.232  -2.045   0.113   0.043   0.844
E R  1MAHA                133    0.417  95.447   0.191   0.199   0.152   0.649
B F  1MAHA                134    0.262  52.583   0.339   0.199   0.152   0.649
B L  1MAHA                135    0.048   8.862  -0.568   0.307   0.165   0.527
B A  1MAHA                136    0.034   3.725  -0.845   0.307   0.165   0.527
E Q  1MAHA                137    0.509  90.907   0.787   0.199   0.152   0.649
E V  1MAHA                138    0.502  77.204  -0.139   0.053   0.043   0.903
E E  1MAHA                139    0.397  69.356   0.226   0.005   0.015   0.979
E G  1MAHA                140    0.321  25.278  -0.438   0.005   0.045   0.951
B A  1MAHA                141    0.036   3.934   0.450   0.002   0.816   0.182
B V  1MAHA                142    0.033   5.134  -0.042   0.001   0.959   0.040
B L  1MAHA                143    0.026   4.706   1.354   0.000   0.983   0.017
B V  1MAHA                144    0.019   2.843   1.399   0.000   0.983   0.017
B S  1MAHA                145    0.042   4.934   0.773   0.001   0.959   0.040
B M  1MAHA                146    0.037   7.424   0.116   0.002   0.816   0.182
B N  1MAHA                147    0.072  10.555  -0.312   0.021   0.451   0.528
B Y  1MAHA                148    0.094  20.045   0.305   0.064   0.216   0.721
B R  1MAHA                149    0.105  24.114  -0.447   0.113   0.087   0.800
B V  1MAHA                150    0.044   6.747   0.452   0.191   0.086   0.723
B G  1MAHA                151    0.073   5.745   0.202   0.113   0.087   0.800
B T  1MAHA                152    0.052   7.268  -1.123   0.113   0.087   0.800
B F  1MAHA                153    0.048   9.734  -0.744   0.118   0.150   0.732
B G  1MAHA                154    0.043   3.368  -2.087   0.066   0.296   0.638
B F  1MAHA                155    0.072  14.551   0.011   0.066   0.296   0.638
B L  1MAHA                156    0.057  10.418  -0.467   0.064   0.216   0.721
B A  1MAHA                157    0.166  18.271  -0.431   0.056   0.142   0.802
B L  1MAHA                158    0.112  20.489  -0.490   0.052   0.084   0.864
E P  1MAHA                159    0.543  76.981  -0.322   0.053   0.043   0.903
E G  1MAHA                160    0.585  46.055  -1.382   0.058   0.017   0.925
B S  1MAHA                161    0.300  35.160  -0.932   0.053   0.043   0.903
E R  1MAHA                162    0.650 148.919  -0.985   0.053   0.043   0.903
E E  1MAHA                163    0.651 113.695  -1.360   0.058   0.017   0.925
B A  1MAHA                164    0.171  18.877  -0.933   0.053   0.043   0.903
E P  1MAHA                165    0.408  57.867  -0.394   0.053   0.043   0.903
B G  1MAHA                166    0.216  17.007  -1.221   0.113   0.043   0.844
B N  1MAHA                167    0.093  13.674  -1.598   0.115   0.016   0.868
B V  1MAHA                168    0.147  22.548   0.241   0.339   0.016   0.645
B G  1MAHA                169    0.069   5.422  -1.211   0.354   0.048   0.598
B L  1MAHA                170    0.046   8.349   0.008   0.717   0.014   0.269
B L  1MAHA                171    0.157  28.765   1.328   0.938   0.007   0.055
B D  1MAHA                172    0.051   7.277  -0.173   0.923   0.002   0.076
B Q  1MAHA                173    0.023   4.126   1.219   0.970   0.001   0.030
B R  1MAHA                174    0.108  24.663   0.451   0.988   0.000   0.012
B L  1MAHA                175    0.075  13.824   0.686   0.988   0.000   0.012
B A  1MAHA                176    0.017   1.851   0.955   0.988   0.000   0.012
B L  1MAHA                177    0.038   6.939   0.406   0.970   0.001   0.030
E Q  1MAHA                178    0.355  63.439   2.057   0.970   0.001   0.030
B W  1MAHA                179    0.059  14.238   0.765   0.970   0.001   0.030
B V  1MAHA                180    0.019   2.905   1.252   0.970   0.001   0.030
E Q  1MAHA                181    0.298  53.169   1.085   0.923   0.002   0.076
E E  1MAHA                182    0.506  88.346   1.450   0.923   0.002   0.076
B N  1MAHA                183    0.089  13.074   1.108   0.858   0.002   0.139
B I  1MAHA                184    0.032   5.883   0.689   0.858   0.002   0.139
E A  1MAHA                185    0.464  51.144   0.431   0.782   0.003   0.216
E A  1MAHA                186    0.472  52.058  -0.466   0.622   0.015   0.363
B F  1MAHA                187    0.091  18.264  -0.274   0.339   0.016   0.645
B G  1MAHA                188    0.279  21.989  -2.083   0.058   0.017   0.925
B G  1MAHA                189    0.181  14.213  -2.616   0.018   0.019   0.964
B D  1MAHA                190    0.237  34.108   0.148   0.018   0.019   0.964
B P  1MAHA                191    0.291  41.321  -0.702   0.018   0.019   0.964
E M  1MAHA                192    0.573 114.577  -0.400   0.018   0.047   0.935
B S  1MAHA                193    0.204  23.874   1.096   0.021   0.279   0.699
B V  1MAHA                194    0.018   2.828   1.206   0.021   0.756   0.223
B T  1MAHA                195    0.048   6.644   0.494   0.011   0.918   0.071
B L  1MAHA                196    0.019   3.516   0.835   0.011   0.918   0.071
B F  1MAHA                197    0.044   8.751   0.291   0.018   0.846   0.136
B G  1MAHA                198    0.027   2.156  -0.803   0.003   0.718   0.279
B E  1MAHA                199    0.072  12.543  -0.363   0.021   0.451   0.528
B S  1MAHA                200    0.040   4.711  -2.370   0.191   0.086   0.723
B A  1MAHA                201    0.043   4.772  -0.301   0.782   0.003   0.216
B G  1MAHA                202    0.030   2.337  -1.191   0.923   0.002   0.076
B A  1MAHA                203    0.023   2.579  -0.519   0.923   0.002   0.076
B A  1MAHA                204    0.048   5.268  -1.384   0.938   0.007   0.055
B S  1MAHA                205    0.027   3.129   0.130   0.938   0.007   0.055
B V  1MAHA                206    0.020   3.013   0.779   0.938   0.007   0.055
B G  1MAHA                207    0.051   4.045  -0.492   0.938   0.007   0.055
B M  1MAHA                208    0.041   8.144   0.201   0.938   0.007   0.055
B H  1MAHA                209    0.035   6.439   0.377   0.938   0.007   0.055
B I  1MAHA                210    0.045   8.380  -0.154   0.782   0.003   0.216
B L  1MAHA                211    0.090  16.442  -0.571   0.622   0.015   0.363
B S  1MAHA                212    0.029   3.340   0.206   0.176   0.004   0.820
E L  1MAHA                213    0.337  61.668  -0.261   0.109   0.005   0.886
E P  1MAHA                214    0.424  60.137  -1.114   0.053   0.005   0.942
B S  1MAHA                215    0.122  14.334  -2.237   0.109   0.005   0.886
E R  1MAHA                216    0.411  94.188   0.317   0.600   0.003   0.397
E S  1MAHA                217    0.383  44.923  -0.503   0.782   0.003   0.216
B L  1MAHA                218    0.084  15.435   0.913   0.858   0.002   0.139
B F  1MAHA                219    0.019   3.894   0.839   0.879   0.010   0.111
B H  1MAHA                220    0.171  31.050   0.461   0.911   0.033   0.057
B R  1MAHA                221    0.160  36.754   0.410   0.911   0.033   0.057
B A  1MAHA                222    0.020   2.160   1.200   0.831   0.044   0.125
B V  1MAHA                223    0.024   3.643   1.091   0.779   0.100   0.120
B L  1MAHA                224    0.033   6.006  -0.610   0.649   0.163   0.188
B Q  1MAHA                225    0.076  13.502  -0.853   0.307   0.165   0.527
B S  1MAHA                226    0.029   3.352  -1.339   0.113   0.087   0.800
B G  1MAHA                227    0.057   4.447  -2.149   0.053   0.043   0.903
B T  1MAHA                228    0.098  13.579  -1.551   0.053   0.043   0.903
B P  1MAHA                229    0.056   8.003  -1.905   0.052   0.084   0.864
B N  1MAHA                230    0.153  22.458  -1.985   0.053   0.043   0.903
B G  1MAHA                231    0.118   9.271  -1.776   0.058   0.017   0.925
B P  1MAHA                232    0.231  32.736  -2.045   0.058   0.017   0.925
B W  1MAHA                233    0.232  55.844  -2.082   0.113   0.043   0.844
B A  1MAHA                234    0.215  23.649  -1.882   0.053   0.043   0.903
B T  1MAHA                235    0.242  33.593  -1.497   0.018   0.088   0.893
E V  1MAHA                236    0.325  49.906  -0.556   0.018   0.047   0.935
E S  1MAHA                237    0.433  50.783  -0.262   0.005   0.015   0.979
E A  1MAHA                238    0.350  38.526  -0.162   0.694   0.003   0.303
E G  1MAHA                239    0.717  56.467   0.092   0.858   0.002   0.139
E E  1MAHA                240    0.420  73.444   0.752   0.923   0.002   0.076
B A  1MAHA                241    0.032   3.537  -0.919   0.970   0.001   0.030
B R  1MAHA                242    0.237  54.365   0.926   0.970   0.001   0.030
E R  1MAHA                243    0.528 120.866   1.293   0.970   0.001   0.030
B R  1MAHA                244    0.138  31.533   1.211   0.970   0.001   0.030
B A  1MAHA                245    0.016   1.774   1.059   0.988   0.000   0.012
E T  1MAHA                246    0.347  48.060   1.806   0.988   0.000   0.012
E L  1MAHA                247    0.364  66.648   2.029   0.988   0.000   0.012
B L  1MAHA                248    0.031   5.713   0.563   0.988   0.000   0.012
B A  1MAHA                249    0.025   2.777  -0.063   0.970   0.001   0.030
E R  1MAHA                250    0.665 152.377   1.732   0.923   0.002   0.076
E L  1MAHA                251    0.438  80.253   0.987   0.858   0.002   0.139
B V  1MAHA                252    0.076  11.712  -0.029   0.406   0.004   0.590
E G  1MAHA                253    0.744  58.553  -0.616   0.016   0.005   0.979
B C  1MAHA                254    0.134  18.884   0.181   0.003   0.003   0.994
E N  1MAHA                255    0.550  80.549   0.274   0.003   0.003   0.994
E D  1MAHA                256    0.337  48.605  -0.221   0.109   0.005   0.886
E T  1MAHA                257    0.659  91.362   0.965   0.858   0.002   0.139
E E  1MAHA                258    0.543  94.775   1.291   0.858   0.002   0.139
B L  1MAHA                259    0.101  18.438   0.762   0.970   0.001   0.030
B I  1MAHA                260    0.092  17.001   0.948   0.970   0.001   0.030
E A  1MAHA                261    0.548  60.346   1.947   0.970   0.001   0.030
B C  1MAHA                262    0.150  21.032   1.069   0.970   0.001   0.030
B L  1MAHA                263    0.029   5.383   0.432   0.923   0.002   0.076
E R  1MAHA                264    0.296  67.853   0.970   0.858   0.002   0.139
E T  1MAHA                265    0.732 101.570   1.573   0.600   0.003   0.397
B R  1MAHA                266    0.157  35.907   1.801   0.406   0.004   0.590
E P  1MAHA                267    0.401  56.959   1.069   0.109   0.005   0.886
B A  1MAHA                268    0.072   7.934   0.132   0.923   0.002   0.076
E Q  1MAHA                269    0.567 101.302   1.589   0.970   0.001   0.030
E D  1MAHA                270    0.346  49.873   2.258   0.970   0.001   0.030
B L  1MAHA                271    0.018   3.351   1.377   0.970   0.001   0.030
B V  1MAHA                272    0.138  21.211   1.077   0.970   0.001   0.030
E D  1MAHA                273    0.523  75.422   1.742   0.970   0.001   0.030
B H  1MAHA                274    0.071  12.933   0.175   0.923   0.002   0.076
B E  1MAHA                275    0.069  12.002  -0.452   0.923   0.002   0.076
E W  1MAHA                276    0.409  98.389   0.066   0.858   0.002   0.139
E H  1MAHA                277    0.462  83.947   0.274   0.782   0.003   0.216
B V  1MAHA                278    0.074  11.358  -0.943   0.717   0.014   0.269
B L  1MAHA                279    0.253  46.416  -0.614   0.622   0.015   0.363
E P  1MAHA                280    0.563  79.833  -1.495   0.522   0.016   0.462
E Q  1MAHA                281    0.393  70.118  -1.839   0.339   0.016   0.645
E E  1MAHA                282    0.440  76.833  -2.363   0.181   0.016   0.803
E S  1MAHA                283    0.403  47.255  -2.494   0.058   0.017   0.925
B I  1MAHA                284    0.201  37.130  -2.313   0.053   0.043   0.903
B F  1MAHA                285    0.157  31.590  -2.839   0.018   0.088   0.893
B R  1MAHA                286    0.170  38.816  -2.386   0.020   0.205   0.775
B F  1MAHA                287    0.068  13.628  -1.330   0.005   0.262   0.733
B S  1MAHA                288    0.059   6.903  -1.737   0.005   0.336   0.660
B F  1MAHA                289    0.037   7.466  -1.144   0.004   0.514   0.481
B V  1MAHA                290    0.071  10.851  -0.468   0.004   0.514   0.481
B P  1MAHA                291    0.028   4.016  -0.175   0.021   0.451   0.528
B V  1MAHA                292    0.049   7.501  -0.381   0.004   0.420   0.576
B V  1MAHA                293    0.129  19.889   0.071   0.005   0.336   0.660
E D  1MAHA                294    0.338  48.706  -0.191   0.018   0.088   0.893
E G  1MAHA                295    0.367  28.899  -0.863   0.018   0.019   0.964
E D  1MAHA                296    0.466  67.107   0.210   0.052   0.084   0.864
B F  1MAHA                297    0.104  20.933   0.154   0.019   0.141   0.840
B L  1MAHA                298    0.034   6.225   0.686   0.019   0.141   0.840
B S  1MAHA                299    0.248  29.019   0.523   0.018   0.088   0.893
E D  1MAHA                300    0.572  82.382   0.840   0.115   0.016   0.868
E T  1MAHA                301    0.349  48.365   1.063   0.115   0.016   0.868
B P  1MAHA                302    0.053   7.535  -0.251   0.923   0.002   0.076
E E  1MAHA                303    0.291  50.838   0.829   0.970   0.001   0.030
E A  1MAHA                304    0.442  48.708   1.543   0.970   0.001   0.030
B L  1MAHA                305    0.050   9.173   0.228   0.970   0.001   0.030
B I  1MAHA                306    0.094  17.409  -0.310   0.923   0.002   0.076
E N  1MAHA                307    0.556  81.413   0.399   0.782   0.003   0.216
E T  1MAHA                308    0.589  81.694   0.662   0.406   0.004   0.590
E G  1MAHA                309    0.503  39.610  -1.345   0.053   0.005   0.942
E D  1MAHA                310    0.534  76.906  -0.146   0.016   0.005   0.979
B F  1MAHA                311    0.106  21.234  -1.327   0.058   0.017   0.925
E Q  1MAHA                312    0.382  68.154   0.006   0.018   0.047   0.935
E D  1MAHA                313    0.551  79.356  -0.855   0.018   0.019   0.964
B L  1MAHA                314    0.131  24.059   0.746   0.018   0.019   0.964
B Q  1MAHA                315    0.118  21.075  -1.387   0.018   0.047   0.935
B V  1MAHA                316    0.044   6.824   0.280   0.003   0.718   0.279
B L  1MAHA                317    0.016   2.966   0.752   0.011   0.918   0.071
B V  1MAHA                318    0.024   3.720   0.473   0.011   0.918   0.071
B G  1MAHA                319    0.016   1.291  -0.304   0.011   0.918   0.071
B V  1MAHA                320    0.043   6.671   0.206   0.002   0.816   0.182
B V  1MAHA                321    0.020   3.013   0.997   0.021   0.451   0.528
B K  1MAHA                322    0.151  31.102   0.375   0.184   0.043   0.773
B D  1MAHA                323    0.164  23.632  -1.250   0.257   0.016   0.727
B E  1MAHA                324    0.031   5.416  -1.089   0.339   0.016   0.645
B G  1MAHA                325    0.061   4.777  -2.926   0.268   0.043   0.689
B S  1MAHA                326    0.102  11.990  -1.725   0.307   0.165   0.527
B Y  1MAHA                327    0.092  19.682  -0.761   0.307   0.165   0.527
B F  1MAHA                328    0.065  13.106  -0.681   0.307   0.165   0.527
B L  1MAHA                329    0.133  24.334  -0.677   0.386   0.097   0.517
B V  1MAHA                330    0.226  34.675  -1.651   0.386   0.097   0.517
E Y  1MAHA                331    0.325  69.559  -1.542   0.354   0.048   0.598
B G  1MAHA                332    0.281  22.146  -1.510   0.257   0.016   0.727
B V  1MAHA                333    0.292  44.819  -1.300   0.339   0.016   0.645
E P  1MAHA                334    0.540  76.598  -2.005   0.339   0.016   0.645
E G  1MAHA                335    0.446  35.100  -1.509   0.406   0.004   0.590
B F  1MAHA                336    0.245  49.171  -1.537   0.406   0.004   0.590
E S  1MAHA                337    0.543  63.616  -1.455   0.502   0.002   0.495
E K  1MAHA                338    0.510 104.907  -0.984   0.502   0.002   0.495
E D  1MAHA                339    0.280  40.362  -1.195   0.600   0.003   0.397
E N  1MAHA                340    0.310  45.384  -1.049   0.694   0.003   0.303
E E  1MAHA                341    0.601 104.942  -0.284   0.782   0.003   0.216
E S  1MAHA                342    0.342  40.129  -0.690   0.782   0.003   0.216
B L  1MAHA                343    0.120  22.027  -0.662   0.802   0.014   0.185
E I  1MAHA                344    0.366  67.692  -0.657   0.802   0.014   0.185
E S  1MAHA                345    0.453  53.138  -0.502   0.717   0.014   0.269
B R  1MAHA                346    0.223  50.975  -0.470   0.717   0.014   0.269
B A  1MAHA                347    0.194  21.346  -0.896   0.717   0.014   0.269
E Q  1MAHA                348    0.408  72.851  -0.300   0.717   0.014   0.269
B F  1MAHA                349    0.131  26.352  -0.361   0.802   0.014   0.185
B L  1MAHA                350    0.149  27.319  -0.800   0.802   0.014   0.185
B A  1MAHA                351    0.235  25.941  -0.766   0.717   0.014   0.269
B G  1MAHA                352    0.150  11.789  -1.286   0.717   0.014   0.269
B V  1MAHA                353    0.083  12.726  -0.428   0.717   0.014   0.269
E R  1MAHA                354    0.420  96.272  -0.437   0.717   0.014   0.269
E I  1MAHA                355    0.387  71.576  -1.081   0.522   0.016   0.462
B G  1MAHA                356    0.213  16.795  -1.741   0.430   0.016   0.555
B V  1MAHA                357    0.157  24.208  -0.295   0.339   0.016   0.645
E P  1MAHA                358    0.541  76.754  -0.992   0.257   0.016   0.727
E Q  1MAHA                359    0.578 103.159  -1.368   0.321   0.003   0.675
B A  1MAHA                360    0.208  22.889  -1.011   0.321   0.003   0.675
E S  1MAHA                361    0.312  36.613  -0.418   0.600   0.003   0.397
E D  1MAHA                362    0.630  90.725  -0.460   0.858   0.002   0.139
E L  1MAHA                363    0.361  66.172   0.203   0.923   0.002   0.076
B A  1MAHA                364    0.063   6.943  -0.712   0.923   0.002   0.076
B A  1MAHA                365    0.178  19.594   0.008   0.970   0.001   0.030
E E  1MAHA                366    0.530  92.574   0.665   0.970   0.001   0.030
B A  1MAHA                367    0.138  15.208   0.282   0.970   0.001   0.030
B V  1MAHA                368    0.040   6.148   0.393   0.970   0.001   0.030
B V  1MAHA                369    0.198  30.402   0.141   0.923   0.002   0.076
E L  1MAHA                370    0.398  72.929  -0.183   0.858   0.002   0.139
B H  1MAHA                371    0.151  27.540  -0.624   0.858   0.002   0.139
B Y  1MAHA                372    0.098  20.857  -0.435   0.694   0.003   0.303
E T  1MAHA                373    0.304  42.206  -1.608   0.502   0.002   0.495
E D  1MAHA                374    0.530  76.344  -2.006   0.339   0.016   0.645
E W  1MAHA                375    0.303  72.799  -1.742   0.176   0.004   0.820
E L  1MAHA                376    0.417  76.426  -1.971   0.109   0.005   0.886
E H  1MAHA                377    0.525  95.407  -1.610   0.053   0.005   0.942
E P  1MAHA                378    0.510  72.426  -1.948   0.053   0.005   0.942
E E  1MAHA                379    0.607 106.060  -2.200   0.053   0.005   0.942
E D  1MAHA                380    0.425  61.243  -1.027   0.053   0.005   0.942
E P  1MAHA                381    0.293  41.520  -1.194   0.782   0.003   0.216
E T  1MAHA                382    0.519  71.985  -0.470   0.923   0.002   0.076
E H  1MAHA                383    0.434  79.036   0.644   0.923   0.002   0.076
B L  1MAHA                384    0.069  12.579   0.033   0.923   0.002   0.076
B R  1MAHA                385    0.106  24.182   0.546   0.970   0.001   0.030
E D  1MAHA                386    0.446  64.283   1.238   0.970   0.001   0.030
B A  1MAHA                387    0.146  16.133   0.325   0.988   0.000   0.012
B M  1MAHA                388    0.025   5.083   0.586   0.988   0.000   0.012
B S  1MAHA                389    0.077   8.989  -0.074   0.988   0.000   0.012
E A  1MAHA                390    0.258  28.465   0.788   0.970   0.001   0.030
B V  1MAHA                391    0.036   5.564  -0.135   0.975   0.003   0.022
B V  1MAHA                392    0.081  12.511   0.092   0.879   0.010   0.111
B G  1MAHA                393    0.067   5.257  -1.229   0.717   0.014   0.269
B D  1MAHA                394    0.071  10.274  -0.840   0.430   0.016   0.555
B H  1MAHA                395    0.093  16.844   0.120   0.455   0.046   0.498
B N  1MAHA                396    0.096  14.040  -0.060   0.578   0.229   0.194
B V  1MAHA                397    0.040   6.102  -0.560   0.478   0.309   0.214
B V  1MAHA                398    0.074  11.374  -0.447   0.649   0.163   0.188
B C  1MAHA                399    0.052   7.245   0.436   0.605   0.105   0.290
B P  1MAHA                400    0.052   7.379  -0.723   0.717   0.014   0.269
B V  1MAHA                401    0.043   6.548  -0.381   0.938   0.007   0.055
B A  1MAHA                402    0.156  17.180   0.357   0.970   0.001   0.030
B Q  1MAHA                403    0.152  27.165   0.572   0.970   0.001   0.030
B L  1MAHA                404    0.042   7.690  -0.086   0.970   0.001   0.030
B A  1MAHA                405    0.044   4.838  -0.275   0.970   0.001   0.030
E G  1MAHA                406    0.326  25.680   0.888   0.970   0.001   0.030
B R  1MAHA                407    0.156  35.678   0.314   0.970   0.001   0.030
B L  1MAHA                408    0.104  18.987   0.189   0.970   0.001   0.030
B A  1MAHA                409    0.190  20.949  -0.452   0.923   0.002   0.076
E A  1MAHA                410    0.639  70.473   0.456   0.782   0.003   0.216
E Q  1MAHA                411    0.375  66.957  -0.835   0.406   0.004   0.590
E G  1MAHA                412    0.689  54.209  -1.262   0.016   0.005   0.979
B A  1MAHA                413    0.194  21.379  -0.803   0.005   0.015   0.979
E R  1MAHA                414    0.381  87.249   0.181   0.004   0.138   0.858
B V  1MAHA                415    0.045   6.840  -0.300   0.003   0.718   0.279
B Y  1MAHA                416    0.064  13.570  -0.174   0.001   0.900   0.099
B A  1MAHA                417    0.066   7.284   0.169   0.001   0.959   0.040
B Y  1MAHA                418    0.027   5.749   0.847   0.001   0.959   0.040
B I  1MAHA                419    0.082  15.225  -0.133   0.001   0.900   0.099
B F  1MAHA                420    0.027   5.359   0.674   0.002   0.816   0.182
B E  1MAHA                421    0.103  18.081  -1.159   0.023   0.655   0.322
B H  1MAHA                422    0.101  18.426   0.197   0.004   0.616   0.381
E R  1MAHA                423    0.365  83.631   0.444   0.005   0.336   0.660
B A  1MAHA                424    0.122  13.400  -1.326   0.018   0.047   0.935
E S  1MAHA                425    0.633  74.141  -1.716   0.018   0.019   0.964
E T  1MAHA                426    0.439  60.862  -2.007   0.018   0.019   0.964
B L  1MAHA                427    0.278  50.865  -2.270   0.018   0.019   0.964
E T  1MAHA                428    0.530  73.442  -2.503   0.018   0.019   0.964
E W  1MAHA                429    0.350  84.271  -1.614   0.018   0.019   0.964
E P  1MAHA                430    0.400  56.817  -1.459   0.018   0.019   0.964
E L  1MAHA                431    0.449  82.139  -1.157   0.018   0.047   0.935
B W  1MAHA                432    0.256  61.640  -1.589   0.018   0.047   0.935
B M  1MAHA                433    0.144  28.894  -0.755   0.052   0.084   0.864
B G  1MAHA                434    0.086   6.808  -1.527   0.052   0.084   0.864
B V  1MAHA                435    0.018   2.797   0.674   0.113   0.087   0.800
B P  1MAHA                436    0.037   5.194   0.019   0.216   0.235   0.548
B H  1MAHA                437    0.026   4.766   0.816   0.321   0.252   0.427
B G  1MAHA                438    0.026   2.046  -0.407   0.605   0.105   0.290
B Y  1MAHA                439    0.068  14.510  -0.324   0.561   0.047   0.393
B E  1MAHA                440    0.027   4.752   0.327   0.660   0.049   0.291
B I  1MAHA                441    0.017   3.127   1.697   0.751   0.050   0.199
B E  1MAHA                442    0.068  11.827   0.640   0.751   0.050   0.199
B F  1MAHA                443    0.044   8.891   0.083   0.751   0.050   0.199
B I  1MAHA                444    0.022   4.126   0.526   0.751   0.050   0.199
B F  1MAHA                445    0.030   6.061   0.039   0.660   0.049   0.291
B G  1MAHA                446    0.177  13.946  -1.478   0.339   0.016   0.645
B L  1MAHA                447    0.102  18.768  -0.241   0.257   0.016   0.727
B P  1MAHA                448    0.205  29.033  -1.205   0.181   0.016   0.803
B L  1MAHA                449    0.282  51.598  -1.680   0.109   0.005   0.886
B D  1MAHA                450    0.239  34.454  -1.988   0.053   0.005   0.942
E P  1MAHA                451    0.498  70.737  -1.907   0.053   0.005   0.942
E S  1MAHA                452    0.665  77.973  -1.919   0.016   0.005   0.979
E L  1MAHA                453    0.300  54.967  -0.697   0.016   0.005   0.979
E N  1MAHA                454    0.649  95.072  -1.076   0.005   0.015   0.979
B Y  1MAHA                455    0.252  53.831  -0.696   0.005   0.015   0.979
E T  1MAHA                456    0.437  60.612   0.005   0.003   0.003   0.994
E T  1MAHA                457    0.695  96.438   0.322   0.694   0.003   0.303
E E  1MAHA                458    0.501  87.490   0.783   0.858   0.002   0.139
B E  1MAHA                459    0.064  11.163  -1.095   0.923   0.002   0.076
E R  1MAHA                460    0.307  70.349   1.491   0.970   0.001   0.030
E I  1MAHA                461    0.442  81.733   1.864   0.970   0.001   0.030
B F  1MAHA                462    0.038   7.607   0.449   0.970   0.001   0.030
B A  1MAHA                463    0.030   3.361   0.248   0.970   0.001   0.030
E Q  1MAHA                464    0.427  76.316   1.621   0.988   0.000   0.012
B R  1MAHA                465    0.213  48.754   1.618   0.988   0.000   0.012
B L  1MAHA                466    0.032   5.804   0.356   0.988   0.000   0.012
B M  1MAHA                467    0.090  18.029   0.816   0.988   0.000   0.012
E K  1MAHA                468    0.395  81.334   0.791   0.988   0.000   0.012
B Y  1MAHA                469    0.098  20.964   0.981   0.988   0.000   0.012
B W  1MAHA                470    0.041   9.957   0.461   0.988   0.000   0.012
B T  1MAHA                471    0.073  10.181   0.149   0.988   0.000   0.012
B N  1MAHA                472    0.171  25.093   0.153   0.970   0.001   0.030
B F  1MAHA                473    0.044   8.751   0.504   0.975   0.003   0.022
B A  1MAHA                474    0.027   3.019   0.310   0.938   0.007   0.055
E R  1MAHA                475    0.321  73.440   1.161   0.802   0.014   0.185
E T  1MAHA                476    0.308  42.761  -0.038   0.430   0.016   0.555
B G  1MAHA                477    0.110   8.649  -1.302   0.058   0.017   0.925
B D  1MAHA                478    0.175  25.145  -0.380   0.018   0.019   0.964
B P  1MAHA                479    0.094  13.395  -1.726   0.018   0.019   0.964
E N  1MAHA                480    0.381  55.822  -0.422   0.003   0.003   0.994
E D  1MAHA                481    0.389  56.084  -0.974   0.018   0.019   0.964
E P  1MAHA                482    0.648  91.909  -1.641   0.018   0.019   0.964
E R  1MAHA                483    0.495 113.332  -0.970   0.018   0.019   0.964
E D  1MAHA                484    0.510  73.462  -1.956   0.018   0.019   0.964
E S  1MAHA                485    0.662  77.551  -1.698   0.018   0.019   0.964
E K  1MAHA                486    0.539 110.913  -1.068   0.018   0.019   0.964
E S  1MAHA                487    0.381  44.653  -0.620   0.005   0.045   0.951
B P  1MAHA                488    0.276  39.179  -0.004   0.004   0.085   0.910
E Q  1MAHA                489    0.605 108.017   1.444   0.004   0.197   0.799
B W  1MAHA                490    0.047  11.400   0.523   0.004   0.138   0.858
E P  1MAHA                491    0.429  60.875   1.136   0.004   0.197   0.799
E P  1MAHA                492    0.517  73.334   0.532   0.005   0.262   0.733
B Y  1MAHA                493    0.184  39.321   0.121   0.004   0.197   0.799
E T  1MAHA                494    0.364  50.487   0.544   0.004   0.085   0.910
E T  1MAHA                495    0.622  86.216  -0.274   0.018   0.019   0.964
E A  1MAHA                496    0.809  89.108  -0.508   0.005   0.015   0.979
E A  1MAHA                497    0.635  69.999  -0.049   0.005   0.015   0.979
E Q  1MAHA                498    0.325  57.991   0.731   0.004   0.138   0.858
E Q  1MAHA                499    0.361  64.403   1.173   0.004   0.616   0.381
B Y  1MAHA                500    0.114  24.426   0.887   0.001   0.900   0.099
B V  1MAHA                501    0.043   6.532   0.406   0.001   0.959   0.040
B S  1MAHA                502    0.248  29.101   0.415   0.001   0.959   0.040
B L  1MAHA                503    0.034   6.317   0.110   0.002   0.816   0.182
E N  1MAHA                504    0.329  48.166  -0.212   0.004   0.197   0.799
E L  1MAHA                505    0.337  61.613  -0.562   0.005   0.015   0.979
E K  1MAHA                506    0.745 153.329   0.293   0.018   0.019   0.964
E P  1MAHA                507    0.543  77.066  -0.859   0.018   0.047   0.935
B L  1MAHA                508    0.267  48.851  -0.507   0.064   0.216   0.721
E E  1MAHA                509    0.457  79.890   0.084   0.021   0.451   0.528
E V  1MAHA                510    0.287  44.097  -0.453   0.022   0.552   0.426
E R  1MAHA                511    0.346  79.211   0.009   0.005   0.336   0.660
E R  1MAHA                512    0.600 137.423  -1.184   0.004   0.197   0.799
E G  1MAHA                513    0.464  36.540  -1.075   0.018   0.047   0.935
B L  1MAHA                514    0.276  50.462  -0.641   0.058   0.017   0.925
E R  1MAHA                515    0.369  84.524   0.582   0.522   0.016   0.462
E A  1MAHA                516    0.480  52.940   0.885   0.858   0.002   0.139
E Q  1MAHA                517    0.532  94.926   0.970   0.858   0.002   0.139
B T  1MAHA                518    0.107  14.827   0.909   0.923   0.002   0.076
B C  1MAHA                519    0.081  11.400   1.432   0.970   0.001   0.030
E A  1MAHA                520    0.516  56.885   1.809   0.970   0.001   0.030
B F  1MAHA                521    0.129  25.910   0.794   0.970   0.001   0.030
B W  1MAHA                522    0.022   5.387   1.233   0.988   0.000   0.012
E N  1MAHA                523    0.391  57.257   1.635   0.970   0.001   0.030
E R  1MAHA                524    0.556 127.324   1.610   0.970   0.001   0.030
B F  1MAHA                525    0.083  16.638   1.197   0.970   0.001   0.030
B L  1MAHA                526    0.065  11.865   1.201   0.970   0.001   0.030
E P  1MAHA                527    0.602  85.410   1.676   0.923   0.002   0.076
E K  1MAHA                528    0.469  96.453   1.583   0.923   0.002   0.076
B L  1MAHA                529    0.109  19.921   0.561   0.858   0.002   0.139
E L  1MAHA                530    0.316  57.915   0.786   0.782   0.003   0.216
E S  1MAHA                531    0.702  82.298   0.641   0.600   0.003   0.397
E A  1MAHA                532    0.434  47.805  -0.415   0.321   0.003   0.675
E T  1MAHA                533    0.735 101.972  -0.972   0.003   0.003   0.994

Explain the output.  Go back.
```

---
